# Supplementary material for: Adiabatic quantum state transfer in a semiconductor quantum-dot spin chain
Source: Nat Commun. 2021 Apr 12;12:2156. doi: 10.1038/s41467-021-22416-5 (PMC8042124; doi:10.1038/s41467-021-22416-5)
Supplement: Supplementary file 1 — Supplementary Information [file 41467_2021_22416_MOESM1_ESM.pdf]

Supplementary Information for
Adiabatic quantum state transfer in a semiconductor quantum-dot spin chain

Yadav P. Kandel,[1] Haifeng Qiao,[1] Saeed Fallahi,[2,3] Geoffrey C. Gardner,[3,4] Michael J. Manfra,[2,3,4,5] and John M. Nichol[1,6]

[1]*Department of Physics and Astronomy, University of Rochester, Rochester, NY, 14627 USA*

[2]*Department of Physics and Astronomy, Purdue University, West Lafayette, IN, 47907 USA*

[3]*Birck Nanotechnology Center, Purdue University, West Lafayette, IN, 47907 USA*

[4]*School of Materials Engineering, Purdue University, West Lafayette, IN, 47907 USA*

[5]*School of Electrical and Computer Engineering, Purdue University, West Lafayette, IN, 47907 USA*

[6]*Corresponding author: john.nichol@rochester.edu*

Figure in the main text	Hyperfine field (MHz)	Hyperfine field noise (MHz)
3(c)	[6.50, -8.00, 60.00, -30.00]	[0.10, 0.10, 0.10, 0.10]
3(d)	[36.70, 10.10, -22.10, 16.20]	[0.10, 0.10, 0.10, 0.10]
4(c)	[-17.54, -16.12, -1.84, 31.92]	[0.10, 0.10, 0.10, 0.10]
4(d)	[-10.52, -17.55, 3.65, 24.11]	[0.10, 0.10, 0.10, 0.10]
5(c)-(d)	[22.00, 0.00, 20.00, 70.00]	[10.00, 10.00, 10.00, 10.00]

Supplementary Table I. Specific values of the hyperfine field and noise at each dot location used in different simulations.

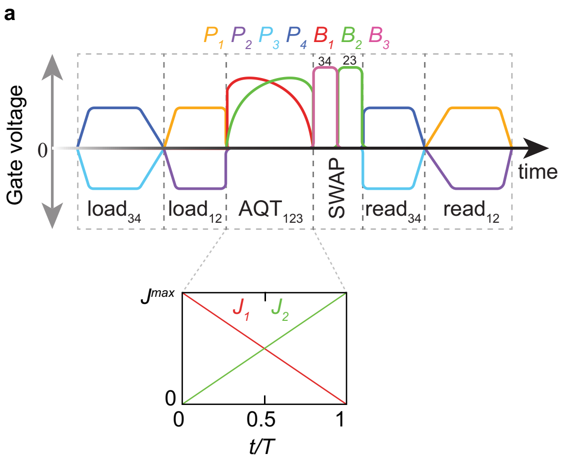

Supplementary Figure 1. Schematic of pulse diagrams for the AQT experiments. (a) Pulse timing diagram corresponding to Fig. 3(a) in the main text. (b) Pulse timing diagram corresponding to the Fig. 4(a) in the main text. Pulses for the modified method to project a two-electron state onto the singlet/triplet (ST) basis are omitted for clarity.

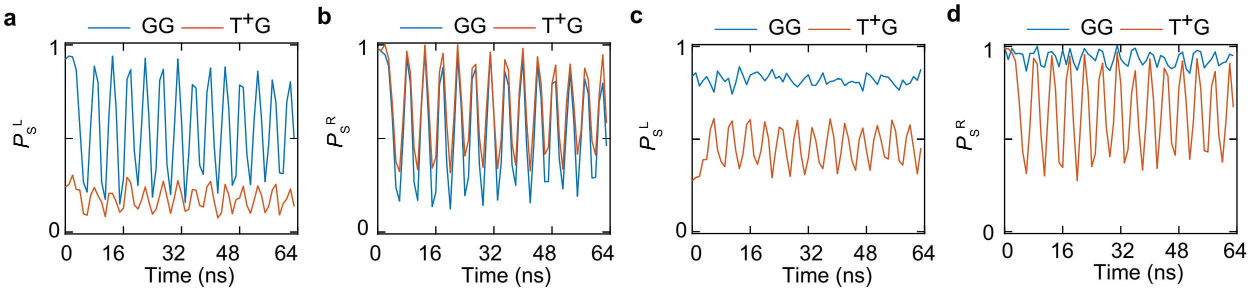

Supplementary Figure 2. Exchange oscillations of spins 2-3 with different load conditions measured on (a, c) the left side and (b, d) the right side. The measurements of panels (a-b) were interleaved together and averaged for 256 single shot measurements. The measurements of panels (c-d) were also interleaved. In panels (a-b), prominent oscillations in the case of an GG initialization imply $f = +1$. Prominent oscillations in P_S^R associated with the T^+G load prove that the ground-state spin orientation in dots 3-4 is $|\downarrow\uparrow\rangle$. From this, we can also infer that the ground state spin configuration in dots 1-2 is $|\downarrow\uparrow\rangle$. Panels (c-d) show a similar data set to panels (a-b), but these data were taken at a different time with a different hyperfine configuration. The oscillations in P_S^R associated with the T^+G initialization, and the absence of oscillations associated with the GG initialization, imply that the ground-state spin configuration of the spin chain is $|\uparrow\downarrow\downarrow\uparrow\rangle$, and $f = -1$.

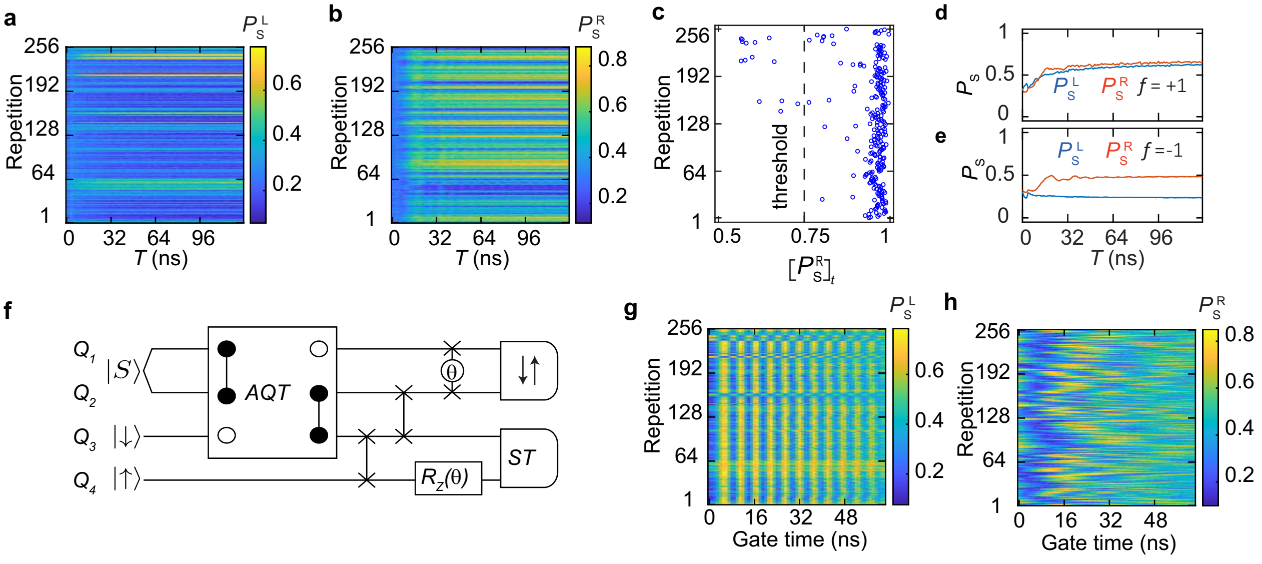

Supplementary Figure 3. (a-b) Data for all repetitions of the experiment described in Fig. 3 in the main text. The line-to-line variations are caused by hyperfine fluctuations, which affect the AQT and the SWAP gates. (c) Time-averaged right-side singlet return probabilities $\left[P_S^R\right]_t$ associated with the evolution of spins 2-3 under the exchange coupling after GG initialization. These measurements were used to determine f. (d-e) Averages over all repetitions of the data in (a-b) corresponding to $f = \pm 1$. (f) Circuit diagram for the verification experiments. The spins are initialized in the state $|S_{12}\uparrow_3\downarrow_4\rangle$ or $|S_{12}\downarrow_3\uparrow_4\rangle$. After the AQT and SWAP operations, spins 1-2 evolve under exchange and spins $3-4$ evolve under ΔB_{34}^z for variable amounts of time. (g-h) Results of the verification experiments. The exchange oscillations in (g) and the singlet-triplet oscillations in (h) provide further evidence of the success of the AQT. In (g), the phase of the oscillations is opposite for $f = \pm 1$, as expected. The frequency of the singlet-triplet oscillations in (h) changes between repetitions because of the fluctuating ΔB_{34}^z. The main experiment, the verification experiment, and measurements to monitor the spin ground were interleaved in time. In panels (a), (b), (g), and (h), each line is averaged over 512 single shot measurements.

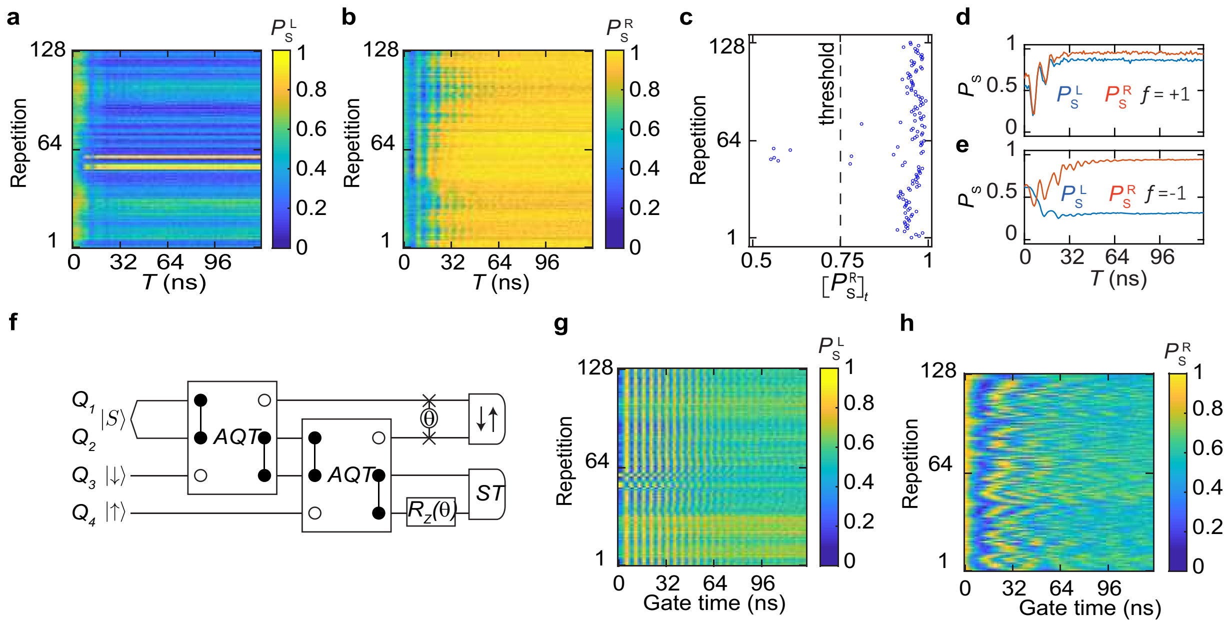

Supplementary Figure 4. (a-b) Data for all repetitions of the AQT cascade experiment described in Fig. 4 in the main text. (c) Time-averaged right-side singlet return probabilities $\left[P_S^R\right]_t$ associated with the evolution of spins 2-3 under the exchange coupling after GG initialization. These measurements were used to determine f. (d-e) Averages over all repetitions of the data in (a-b) corresponding to $f = \pm 1$. (f) Circuit diagram for the verification experiments. The spins are initialized in the state $|S_{12}\uparrow_3\downarrow_4\rangle$ or $|S_{12}\downarrow_3\uparrow_4\rangle$. After the AQT cascade, spins 1-2 evolve under exchange and spins 3-4 evolve under ΔB_{34}^z for variable amounts of time. (g-h) Results of the verification experiments. The exchange oscillations in (g) and the singlet-triplet oscillations in (h) provide further evidence of the success of the AQT. In (g), the phase of the oscillations is opposite for $f = \pm 1$, as expected. The frequency of the singlet-triplet oscillations in (h) changes between repetitions because of the fluctuating ΔB_{34}^z. The main experiment, the verification experiment, and measurements to monitor the spin ground were interleaved in time. In panels (a), (b), (g), and (h), each line is averaged over 256 single shot measurements.

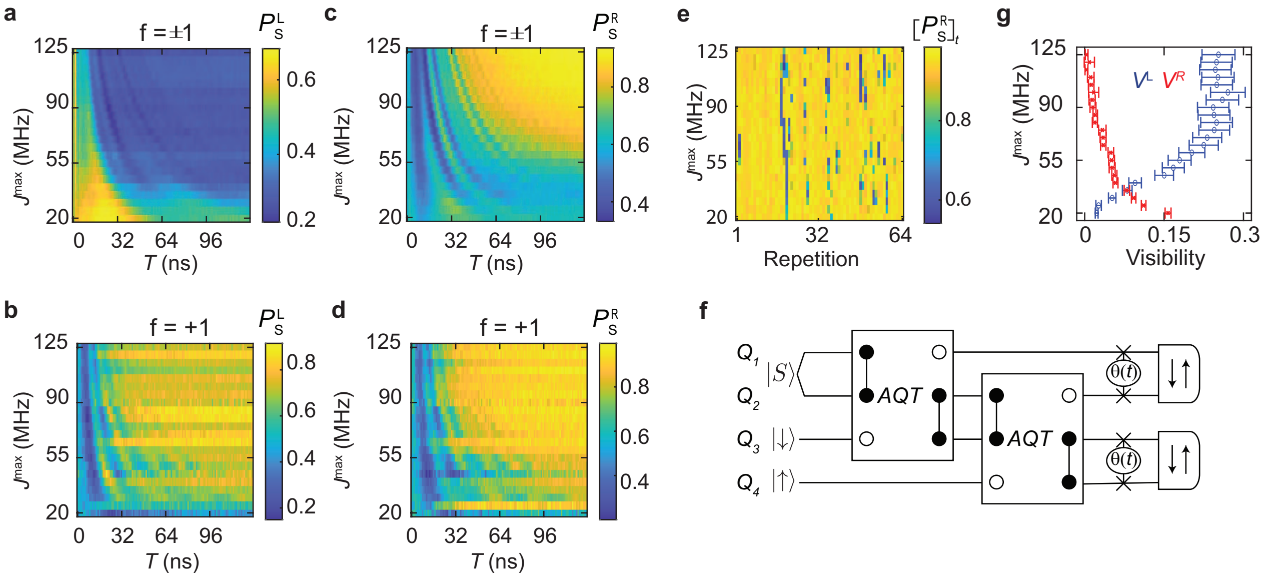

Supplementary Figure 5. Data for the experiment described in Fig. 5 in the main text. (a,c) Raw data for the left and the right sides. Each line is the average of 128 single shot measurements which are repeated 64 times. Out of all those 64 repetitions for 22 line corresponding to 22 different values of the J^{max}, 95% correspond to the $f = -1$ which is presented in the Fig.5 in the main text. (b,d) Post-selected data for the left and the right sides for $f = +1$. (e) Time-averaged right-side singlet return probability $\left[P_S^R\right]_t$ associated with the evolution of spins 2 and 3 under exchange coupling corresponding to GG initialization. We bin the main data set into $f = \pm 1$ cases by thresholding the data according to the measurements of panel (e), as discussed above. In most of the cases f is -1. (f) Quantum circuit diagram to monitor exchange oscillation visibility after the AQT cascade. (g) Exchange oscillation visibility $V^{R/L}$ of the right and the left spins pairs for different J^{max} with $T = 127$ ns for $f = -1$. V^L increases gradually with J^{max} and saturates.

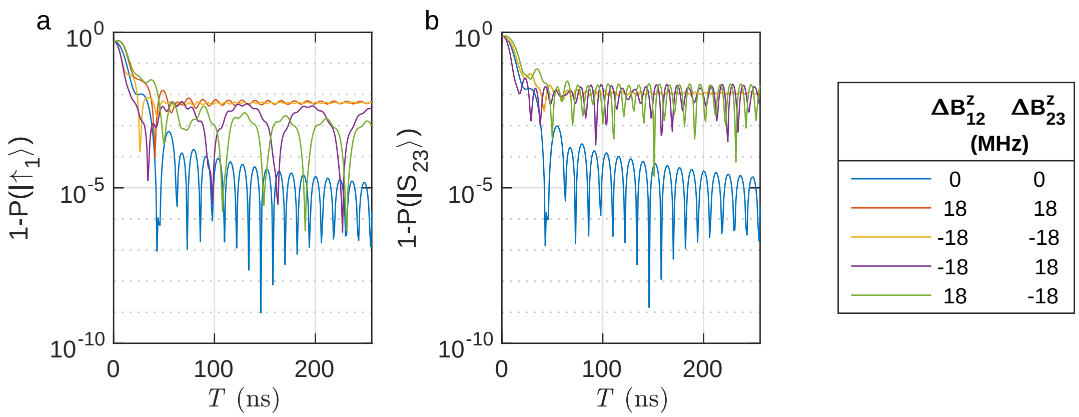

Supplementary Figure 6. Simulated effects of a static magnetic field gradient on AQT fidelity. We simulated the evolution of three-spin chain initialized in the state $|S_{12}\uparrow_3\rangle$. We ramped down the exchange coupling between spins 1 and 2 from 120 MHz to 0 while the exchange coupling between spins 2 and 3 was ramped up from 0 to 120 MHz in a time T. (a) Infidelity of transferring the eigenstate of spin 3 to spin 1 as a function of T. (b) Infidelity of transferring the singlet state from spin 1-2 to 2-3. No magnetic or charge noise was included in these simulations. The simulations show that the AQT fidelity depends sensitively on ΔB^z.

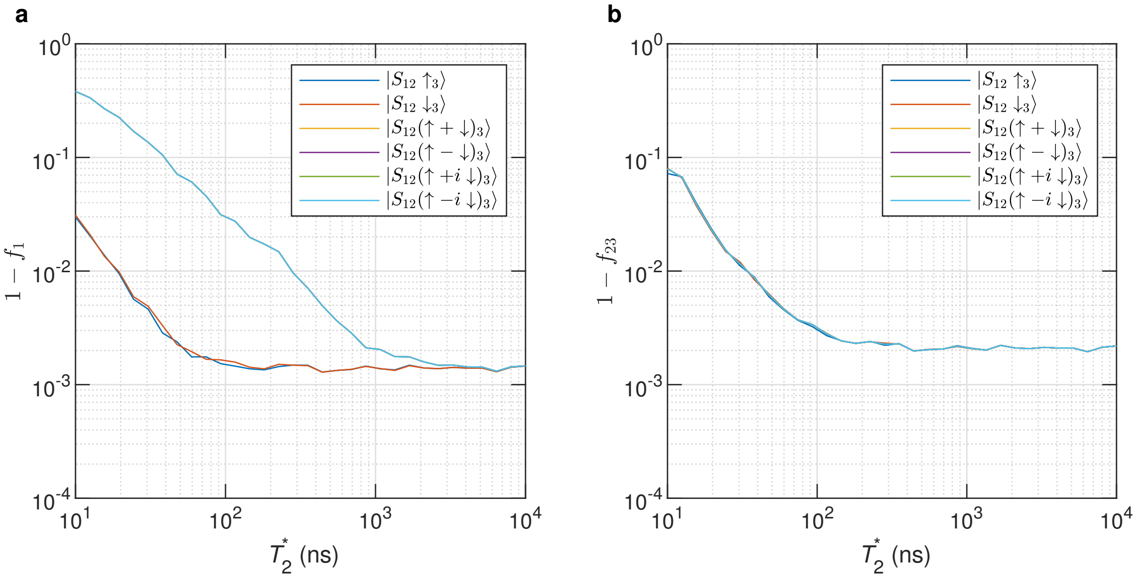

Supplementary Figure 7. Simulated error in the AQT process vs. single-spin T_2^* values for states of the form $|S_{12}\phi_3\rangle$, where $|\phi\rangle$ can be a superposition state. A typical T_2^* value for GaAs is 10-20 ns. For isotopically purified Si, T_2^* can exceed 1 μs. (a) Probability to incorrectly transfer a single-spin state from dot 3 to dot 1. All of the initial states with superposition states of spin 3 have an error similar to the blue curve. (b) Probability to incorrectly transfer the state of dots 1-2 to 2-3. For isotopically purified Si, these simulations suggest that the state-transfer probability can exceed 0.99. State normalization factors have been omitted in the legends.

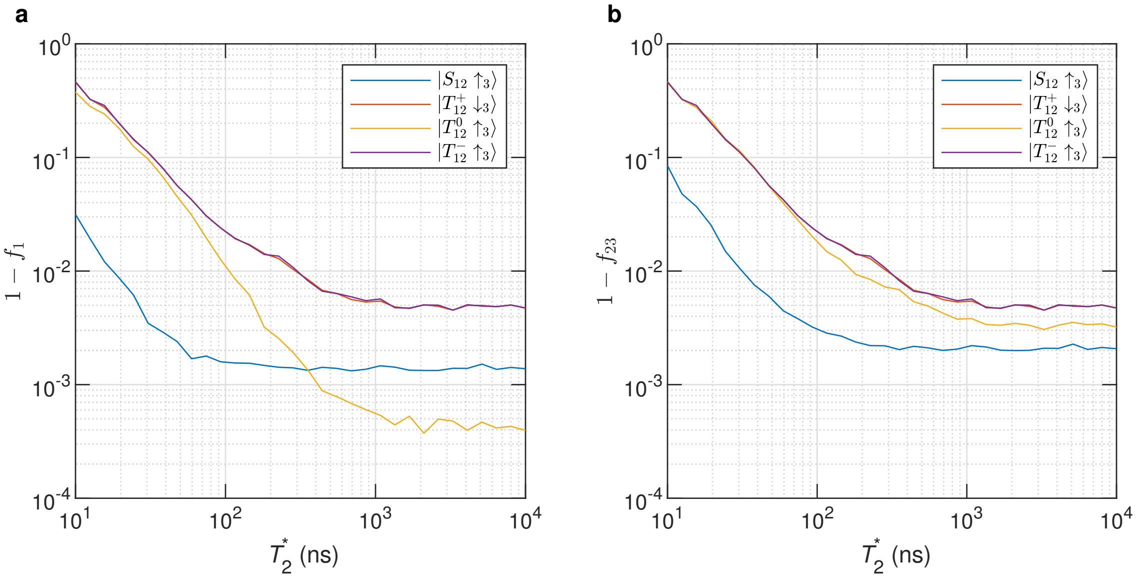

Supplementary Figure 8. Simulated error in the AQT process vs. single-spin T_2^* values for states of the form $|\pi_{12}\phi_3\rangle$, where $|\pi\rangle$ can be a eigenstate of exchange, and $|\phi\rangle$ is a single-spin eigenstate. A typical T_2^* value for GaAs is 10-20 ns. For isotopically purified Si, T_2^* can exceed 1 μs. (a) Probability to incorrectly transfer a single-spin state from dot 3 to dot 1. (b) Probability to incorrectly transfer the state of dots 1-2 to 2-3. For isotopically purified Si, these simulations suggest that the state-transfer probability can exceed 0.99.

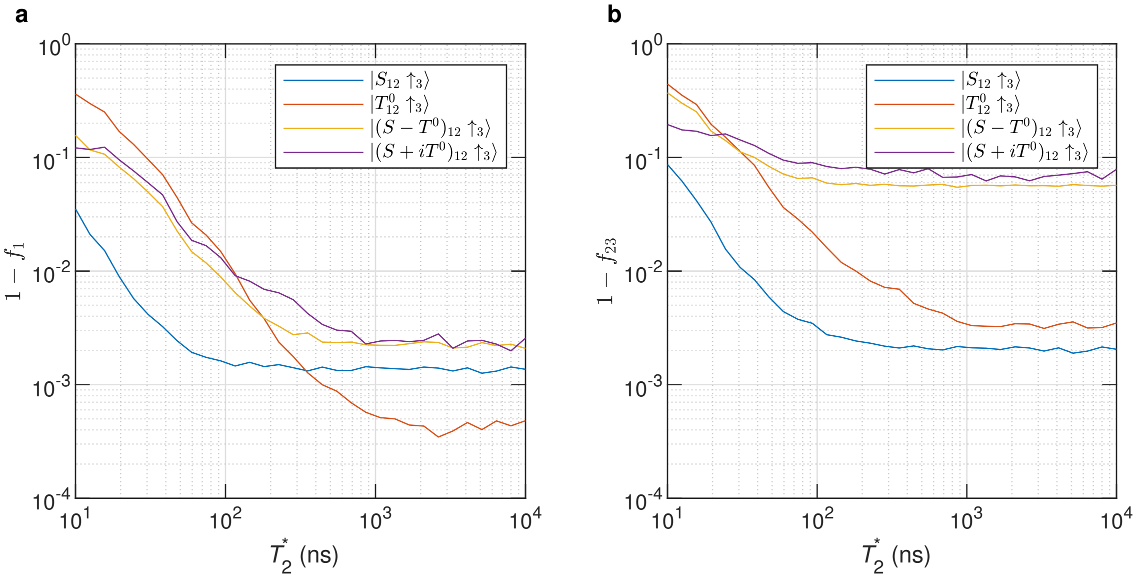

Supplementary Figure 9. Simulated error in the AQT process vs. single-spin T_2^* values for states of the form $|\pi_{12}\uparrow_3\rangle$, where $|\pi\rangle$ can be a superposition of exchange eigenstates. A typical T_2^* value for GaAs is 10-20 ns. For isotopically purified Si, T_2^* can exceed 1 μs. (a) Probability to incorrectly transfer a single-spin state from dot 3 to dot 1. (b) Probability to incorrectly transfer the state of dots 1-2 to 2-3. For isotopically purified Si, these simulations suggest that the single-spin-eigenstate transfer probability can exceed 0.99. State normalization factors have been omitted in the legends

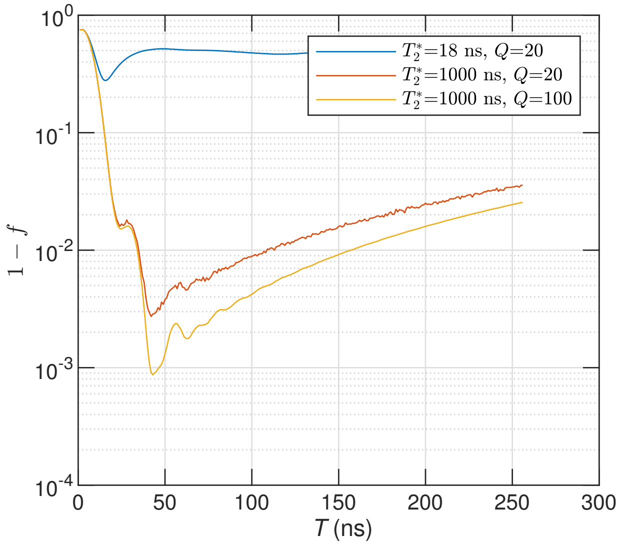

Supplementary Figure 10. Simulated process infidelity for different noise configurations versus Hamiltonian interpolation time T.
